# The Exstrophy-epispadias complex

**DOI:** 10.1186/1750-1172-4-23

**Published:** 2009-10-30

**Authors:** Anne-Karoline Ebert, Heiko Reutter, Michael Ludwig, Wolfgang H Rösch

**Affiliations:** 1 Department of Pediatric Urology, University Medical Center Regensburg, Germany; 2 Department of Human Genetics, University of Bonn, Germany; 3 Department of Neonatology, Childrens' Hospital, University of Bonn, Germany; 4 Department of Clinical Chemistry and Pharmacology, University of Bonn, Germany

## Abstract

Exstrophy-epispadias complex (EEC) represents a spectrum of genitourinary malformations ranging in severity from epispadias (E) to classical bladder exstrophy (CEB) and exstrophy of the cloaca (EC). Depending on severity, EEC may involve the urinary system, musculoskeletal system, pelvis, pelvic floor, abdominal wall, genitalia, and sometimes the spine and anus. Prevalence at birth for the whole spectrum is reported at 1/10,000, ranging from 1/30,000 for CEB to 1/200,000 for EC, with an overall greater proportion of affected males. EEC is characterized by a visible defect of the lower abdominal wall, either with an evaginated bladder plate (CEB), or with an open urethral plate in males or a cleft in females (E). In CE, two exstrophied hemibladders, as well as omphalocele, an imperforate anus and spinal defects, can be seen after birth. EEC results from mechanical disruption or enlargement of the cloacal membrane; the timing of the rupture determines the severity of the malformation. The underlying cause remains unknown: both genetic and environmental factors are likely to play a role in the etiology of EEC. Diagnosis at birth is made on the basis of the clinical presentation but EEC may be detected prenatally by ultrasound from repeated non-visualization of a normally filled fetal bladder. Counseling should be provided to parents but, due to a favorable outcome, termination of the pregnancy is no longer recommended. Management is primarily surgical, with the main aims of obtaining secure abdominal wall closure, achieving urinary continence with preservation of renal function, and, finally, adequate cosmetic and functional genital reconstruction. Several methods for bladder reconstruction with creation of an outlet resistance during the newborn period are favored worldwide. Removal of the bladder template with complete urinary diversion to a rectal reservoir can be an alternative. After reconstructive surgery of the bladder, continence rates of about 80% are expected during childhood. Additional surgery might be needed to optimize bladder storage and emptying function. In cases of final reconstruction failure, urinary diversion should be undertaken. In puberty, genital and reproductive function are important issues. Psychosocial and psychosexual outcome depend on long-term multidisciplinary care to facilitate an adequate quality of life.

## Disease name, synonyms, relevant terms

Exstrophy-epispadias complex (EEC), refers to the whole spectrum

Epispadias (E), penopubic, penile, glandular in males; distal, intermediate, severe in females

Classical bladder exstrophy (CEB)

Exstrophy of the cloaca (EC), often referred as OEIS complex (omphalocele, exstrophy, imperforate anus and spinal defects)

Exstrophy variants like "atypical" forms of the EEC (duplicated exstrophy, covered exstrophy, pseudo-exstrophy).

## Definition

The Exstrophy-epispadias complex (EEC) is the most serious form of abdominal midline malformation. The characteristic defects of the EEC involve the urinary system, the musculoskeletal system, the pelvis, the pelvic floor, the abdominal wall, the genitalia and sometimes the spine and the anus [1]. The EEC covers a spectrum with different severity levels, ranging from epispadias (E) representing the mildest form, with lower and upper fissure, to the full picture of classical bladder exstrophy (CEB), and exstrophy of the cloaca (EC) - often also referred to as OEIS (omphalocele, exstrophy, imperforate anus and spinal defects) complex - as the most severe form. EEC can be subdivided into "classic" or "typical" forms of EEC (E, CEB, and EC) and "atypical" forms of the EEC (duplicated exstrophy, covered exstrophy and pseudo-exstrophy).

## Epidemiology

Varying data have been reported on the incidence of the EEC, especially in respect to various subtypes, different ethnic groups and the male-to-female ratio. Altogether, the combined incidence of the EEC spectrum can be estimated at 1 in 10,000 births. A higher occurrence in males compared to females is observed, ranging from a ratio of 1.5:1 to 6.0:1 [2-5].

For E, the International clearinghouse for birth defects monitoring systems estimated an average rate of 2.4 per 100,000; among the included 148 cases, only four were females [3]. However, it is likely that a proportion of incontinent females with E remain undiagnosed [6]. Taking this into consideration, a recently reported male-to-female ratio of 1.4:1 can be concluded [7]. Even in Europe, the rates have been shown to range from 0.6 per 100,000 in France to 4.7 per 100,000 in Denmark [3]. The highest rate of 8.1 per 100,000 has been observed in Native American Indians, while an incidence of 1 per 100,000 was found for Americans of Asian origin [8].

The reported incidence of CEB varies from 2.1 to 4.0 per 100,000 live births [3,9-12]. CEB seems to occur more frequently in white infants and the incidence varies according to the geographic region, and socioeconomic and insurance status [11]. White, non-Hispanic maternal ethnicity was also found to be associated with CEB in a survey from a period from 1983 to 1999 in the New York State [12]. The results of this study demonstrated that CEB showed a statistically significant linear downward trend by year and summer conception, and male sex was also identified as a possible risk factor. Though Nelson et al. [11] found an almost equal male-to-female ratio for CEB, multiple surveys summarized a ratio of 2.4:1 [4,7,13-15]. In rare cases only, male-to-female ratios as high as 5:1 to 6:1 have been reported [3,5].

EC, with a prevalence ranging from 0.5 to 1 per 200,000 life births [5,10,12,16-20], has been reported more commonly in females [10]. This was confirmed by the New York State survey, finding associated risk factors like preterm birth, low birth weight, multiple births and non-New York City residence [12]. A sex ratio close to unity was found in the series of Boyadjiev et al. [2] and a male-to-female ratio of 2.0:1 was reported by Gambhir et al. [7]. As reviewed by Keppler-Noreuil [21], EC seems to be under-ascertained in still borns, and may therefore have a higher incidence ranging from 1 in 10,000 to 1 in 50,000. In addition, having included terminated EC cases, the prevalence of EC in the State of Iowa was most probably more accurately estimated as occurring in approximately 1 in 27,174 pregnancies [22].

## Clinical description

### Descriptive anatomy

All sections of the EEC have a specific clinical presentation and are obviously recognized right after birth by the pediatrician and the obstetrician.

### Classical bladder exstrophy

CEB is characterized by the evaginated bladder plate of different individual size. Urine is dripping from the ureteric orifices on the bladder surface. The visible bladder mucosa appears reddish at birth and mucosal polyps may be seen on the surface. Delayed closure, however, may lead to further inflammatory or mechanical alterations with signs of mucosal inflammation such as a whitish coating, ulcerations and hyperplastic formations. The paraexstrophic shining thin skin stripes mark the transition junction between the normal skin and squamous metaplastic area. Below the low situated umbilicus, rectus diastasis and small umbilical hernias can be palpated. At the distal end of the triangular edges, the pubic bones can be felt on both sides of the bladder template. Bilateral inguinal hernias are palpable in most patients of both sexes.

### Male genital anatomy in CEB

**Figure 1:**
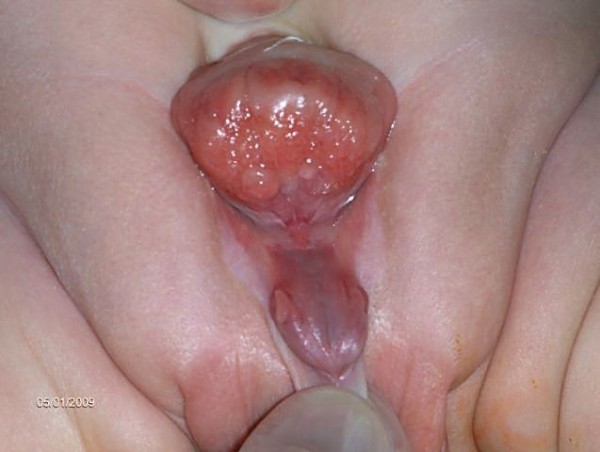
**Male baby with classical bladder exstrophy**.

In male newborns, an open (= epispadic) urethral plate covers the whole dorsum of the penis from the open bladder to the glandular grove. Both corpora cavernosa are located beneath the urethral plate. Careful examination reveals the colliculus seminalis and the ductus ejaculatorii as tiny openings in the area, where the prostate is presumably dorsally located. The penis appears shorter than normal and dorsally curved (Fig. 1). The normal-sized testes are usually located in the scrotum.

### Female genital anatomy in CEB

**Figure 2:**
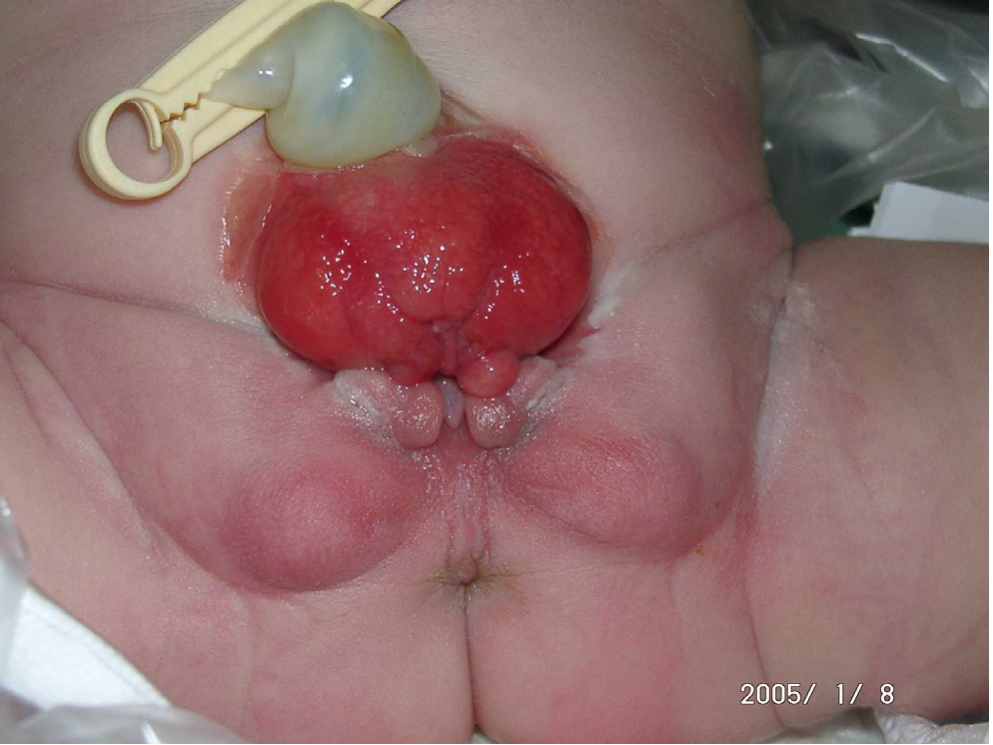
**Female baby with classical bladder exstrophy**.

In females, a completely split clitoris can be seen next to the open urethral plate (Fig. 2). The vaginal opening appears narrow and is placed anteriorly on the perineum. As the anus is ventrally positioned as well, the perineum is shortened.

### Epispadias in both sexes

**Figure 3:**
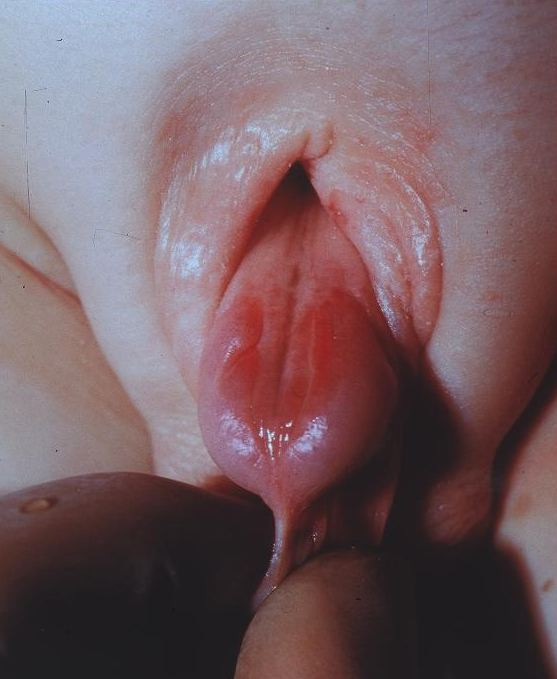
**Male baby with epispadias**.

**Figure 4:**
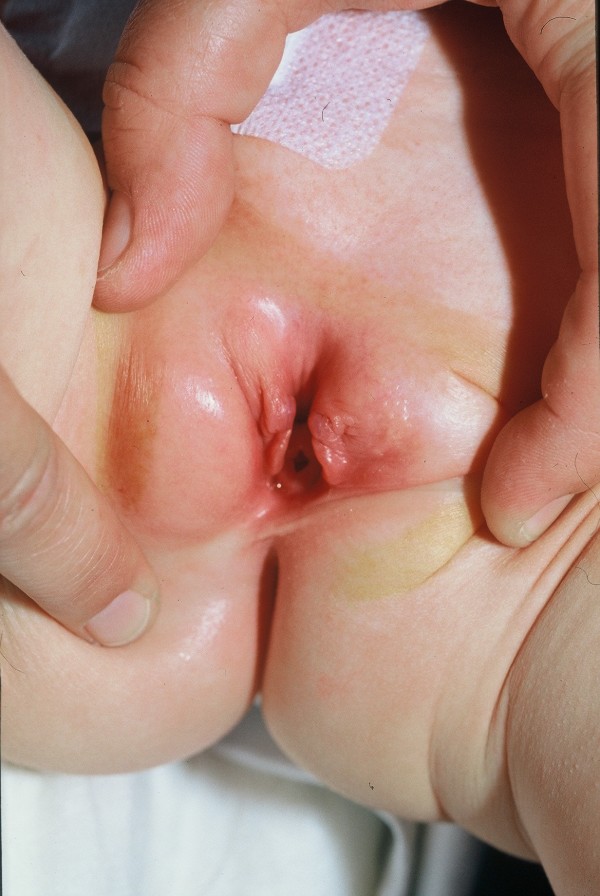
**Female baby with epispadias**.

The E defect in both sexes results from a developmental arrest in terms of non-closure of the urethral plate and additionally in an abnormal dorsal urethral location. Therefore, in males an ectopic meatus or a mucosal strip is found on the penile dorsum (Fig. 3) and in females a variable cleft of the urethra is detected (Fig. 4). According to the meatal location, E is distinguished as either penopubic, penile or glandular in boys. In girls, E is divided into three degrees according to Davis [1], either less severe with a gaping meatus, intermediate or severe with a cleft involving the whole urethra and the bladder neck, additionally displaying bladder mucosal prolapse. Abdominal wall and rectus anatomy, as well as the umbilicus, are completely normally developed. In both sexes symphysis is closed or only a minor symphysis gap is palpable, indicating only minor pelvic and pelvic floor anomalies. Urinary incontinence appears to be the main clinical symptom, due to the degree of involvement of the urinary sphincter. In most distal E, involuntary urine loss is not observed, whereas in complete E urine is dripping permanently through the meatus in both sexes. Due to the sometimes minor clinical abnormalities, distal E might be overlooked at birth, especially in girls. Then diagnosis may be recognized as late as at school age, due to urinary incontinence, resistant to standard treatment.

### Cloacal exstrophy

**Figure 5:**
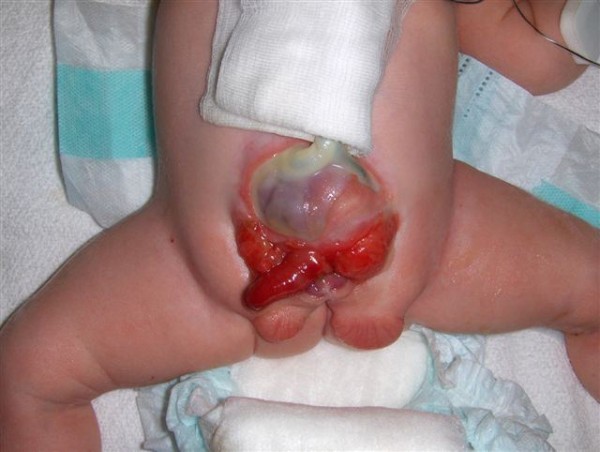
**Male newborn with cloacal bladder exstrophy**.

EC, as a major birth defect, involves several important organ systems. Beside the exstrophy at birth, omphalocele, imperforate anus and spinal defects may be present and mandate immediate surgery. Usually, a foreshortened hindgut or cecum ends between the two exstrophied hemibladders. The orifice of the terminal ileum is located at the everted cecum. The symphysis pubis is widely separated and the pelvis is, in contrast to CEB, often asymmetrically shaped. The genitalia, for instance the penile or clitoral halves, can be located separately on either side of the bladder plates together with the adjacent scrotal or labial part (Fig. 5).

### Exstrophy variants

Exstrophy variants include a clinically inhomogeneous spectrum. The covered exstrophy appears similar to the CEB, just some part of the reddish bladder mucosa may have a roof of skin, for instance. An umbilicus might be at an orthotopic place. Pseudo-exstrophy, however, may be very difficult to define after birth and is therefore often found at older ages. The genitalia look normal, the patients may not have any urinary symptoms and furthermore are completely continent. Clinical inspection may find a rectus diastasis of various degree and only X-rays may demonstrate an open symphysis diastasis, not seldom as an incidental finding.

### Associated anomalies

#### Urological anomalies

In both sexes, urological malformations (*e.g*. ureteropelvic junction obstruction, ectopic pelvic kidney, horseshoe kidney, renal hyo- or agenesis, megaureter, ureteral ectopy and ureterocele) are present in about one third of all EEC cases, predominantly in the EC population [1]. However, a 100% prevalence of bilateral vesicoureteral reflux due to a developmental failure of the ureterovesical junction throughout the EEC spectrum warrants an antireflux procedure with every bladder neck plasty.

#### Spinal and orthopedic anomalies

The incidence of spinal anomalies widely varies within the EEC spectrum. In children born with CEB, spinal anomalies occur in about 7% of cases, whereas a heterogeneous group of congenital spinal anomalies resulting from defective closure of the neural tube early in fetal life and anomalous development of the caudal cell mass can be confirmed with magnetic resonance imaging (MRI) in nearly 100% of EC patients. Therefore, newborn EC patients should have spinal ultrasound and radiographs to define the individual spinal abnormalities ranging from hemivertebra to myelomeningocele. MRI is further recommended in follow-up to identify occult spinal abnormalities predisposing to symptomatic spinal cord tethering. In addition, a neurological component must be kept in mind in EC in respect to bladder function, lower extremity function and erectile capacity [23-25].

Mainly in EC, skeletal and limb anomalies (clubfoot deformities, absence of feet, tibial or fibular deformities, and hip dislocations) are commonly seen [1]. In addition to the clinical examination, a sonographic evaluation of the hip joints is of fundamental impact for all EEC patients. Despite this, there are still no extraordinary reports about hip dysplasia in long-term follow-up of the EEC. Management (even if conservative) should be kept in mind when planning operative reconstruction of the pelvis. Plain pelvic X-ray can be helpful to estimate the dimension of the symphysis gap and the hip localization.

#### Gastrointestinal anomalies

Gastrointestinal tract anomalies are predominantly associated with EC, and will seldom be present in CEB or E. In addition to a common hindgut remnant of varying size, omphaloceles are found in 88-100% of cases in EC. Gastrointestinal malrotation or duplication, as well as short bowel syndrome, can be seen in up to 46% of cases [1]. In about 25% of cases, an either anatomical or functional short bowel syndrome causes absorptive dysfunction. On rare occasions, duodenal atresia and small bowel deletion have been described in EC [1,25]. Thus, the gastrointestinal tract contributes significantly to the morbidity in EC.

#### Gynecological anomalies

In addition to the external female genitalia as described above, the cervix inserts in most cases low down at the superior vaginal wall close to the introitus [1,26]. Anatomy and function of the uterus and the adnexae are normal. However, the pelvic floor and the levator defect, together with the absence of the cardinal ligaments, predisposes women to vaginal or uterine prolapse in as high as 50% of cases. Müllerian anomalies are quite common in EC; respectively, duplication of the vagina and uterus, as well as vaginal agenesia have been reported [1].

## Etiopathogenesis

### Embryology

In 1964 Muecke was the first to report that mechanical disruption or enlargement of the cloacal membrane in chicks prevents the invasion of mesodermal cells along the infraumbilical midline, and thereby results in exstrophy [27]. Based on that, Austin et al. provided evidence that in humans, anomalous overgrowth of the cloacal membrane is associated with bladder exstrophy [28]. Animal models of EC support the idea that abnormal partitioning of the cloacal membrane causes displacement of the genital tubercle and therefore epispadias formation. Accordingly, on the basis of a developmental study of hereditary anorectal malformations in pig embryos, it has been concluded that agenesis of the dorsal part of the cloacal membrane may form the basis of congenital malformations of cloaca-derived orifices such as hypospadias, epispadias, bladder and cloacal exstrophy, double urethra, and cloacal membrane agenesis [29]. Additionally, Thomalla et al. created a hernia defect of the lower abdominal wall of chick embryos by incising the cloacal membrane with a laser [30]. The resulting chicks were born with EC, supporting the idea of premature rupture of the cloacal membrane. The timing of cloacal membrane disruption in this model determined the resulting variant of the EEC, with an earlier disruption (4-6 gestation weeks, before fusion of the urorectal septum to the cloacal membrane) leading to the more severe EC [1]. The authors postulated that EEC would form if cloacal membrane rupture occurred just after the urorectal septum completed its descent (6 weeks), but before the initial formation of the genital tubercle [31-33]. Mechanical obstruction of mesoderm migration to the lower ventral abdominal wall has also been associated with abnormal caudal insertion of the body stalk, resulting in failure of the normal mesodermal interposition in the lower midline [34,35]. In this case, the umbilical cord is directly adjacent to the cloacal membrane/cloaca, and the superficially placed, unstable cloacal membrane/cloaca is prone to rupture. In addition to mechanical disruption, localized alterations in cell death may reduce the ventral mesenchymal cell population and therefore lead to infra-umbilical midline deficiencies in mice, including EEC [35-38]. Another completely different mechanism for the organogenesis of CEB is postulated by the observations of the pelvic development in rabbit embryos, relating bladder and pelvic bone anomalies in temporal-spatial development [39]. This novel mechanism may occur as early as secondary gastrulation without any involvement of the cloacal membrane. In addition, two reports describe the incidental occurrence of EC in chicken embryos subsequent to the administration of nigericin and ochratoxin A [37,40]. Männer and Kluth were able to generate EC in six Leghorns by treating chicken embryos with suramin or trypan blue [41]. The authors saw the abnormal presence of primary large aneurysmatic space-occupying swellings of the dorsal aortae, leading to a thinning of the ventral abdominal wall, finally inducing rupture and exposure of the embryonic cloaca and the allantois [41]. Only one gene, *p63*, apart from causing congenital defects of the extremities and skin, has been shown to completely reproduce human bladder exstrophy in *p63*^-/- ^mice [42]. As noted by Ince et al., female *p63*^-/- ^mice exhibited abnormal genital morphogenesis with hypoplastic genitalia, a single cloacal opening, and persistence of columnar epithelium at lower genital tract sites [43].

### Recurrence risk

The possible role of genetic factors implicated in the expression of EEC is based on the increased recurrence risk for offspring of affected individuals. Among siblings, Ives et al. estimated the recurrence risk to be approximately 1% in non-consanguineous and non-affected parents of CEB cases [5], whereas other reports established a risk of 2.3% [44], 0.8% [2], 0.5% [45] and of 0.3% [46], respectively.

The simplest way to measure genetic effects is through familial risk ratios, defined as the risk to a given type of relative of an affected individual divided by the population prevalence. These risk ratios have been given the notation λ, and specifically λs for the sibling risk ratio, and λo for the offspring risk ratio. Shapiro et al. [46] estimated the risk of recurrence for siblings (s) of European background for isolated CBE as 1 in 275, having surveyed 2,500 CEB families. Based on a prevalence of 3.3:100,000 (~1:30.000) for isolated CEB among populations of European background, the recurrence risk ratio in siblings (λs) has been calculated to be 108 (1:275/1:30.000 ~108) [47]. λS is extensively used to measure familial aggregation of complex diseases, which is defined as the ratio of the risk of disease manifestation, given that one's sibling is affected, as compared with the disease prevalence in the general population. Furthermore, in their series Shapiro et al. [46] described a 400-fold increased risk of CEB in offspring (o) of affected individuals compared to the general population. Likewise, Ludwig et al. concluded, from the review of the available literature, that the relative recurrence risk (s) is 350-500 [48]. While female EEC patients represent the minority of patients, interestingly, only affected females produced affected offspring [46]. Among other explanations, this superficial higher recurrence risk for affected females may be due to a higher genetic liability for EEC (so-called Carter effect) [49].

### Familial recurrences

Although familial occurrence is rare, 30 reported multiplex families support the idea of genetic susceptibility underlying EEC [50]. In most of these families, two members are affected. Only in two families, three affected members of both genders have been described with EEC defects of different severity. Additionally, in a unique Moroccan family, three males (two cousins and a maternal uncle) were affected with CEB [51]. As a conclusion, in rare families the inheritance of EEC may be consistent with autosomal dominant inheritance with reduced penetrance or with an autosomal recessive trait or X-linked transmission [52].

These observations indicate that (a) gene(s) with a major effect on the phenotype exist, though in the majority of cases additional causal factors are necessary for the phenotype to occur. As for other birth defects, a small subgroup of cases may follow Mendelian inheritance whereas in the majority, EEC is inherited as a complex trait with multiple genetic factors (heritable or *de novo *somatic or germline mutations), and complex gene-gene, or gene-environment interactions contributing to its formation.

### Twin studies

Reutter et al. compared concordant rates among EEC twin pairs and established 7.5 times and 5.6 times higher pairwise and probandwise concordant rates, respectively, among monozygotic twins compared to dizygotic twins [47]. The resultant monozygotic/dizygotic twin probandwise concordant rate of 5.6:1 depicts the risk among monozygotic twins of both being affected, which is 5.6 times the risk compared to dizygotic twins. This value, by exceeding 2, indicates that genetic influences from multiple loci may be non-additive, but rather multiplicative and that epistasis (interaction between contributing genes) may exist. This assumption is supported by values obtained for the familial risk ratios: Reutter et al. estimated familial risk ratios for monozygotic twins and dizygotic twins of 4,500 and 600, respectively, resulting in a monozygotic/dizygotic twin ratio of 7.5 [47,53].

### Molecular genetics of the EEC

Cytogenetic and molecular analyses have revealed chromosomal anomalies in 20 EEC patients to date, although none of these appear to be causative. Numerical chromosomal aberrations [47, XXX (observed twice); 47, XXY; 47, XYY; 47,(no sex reported),+18; 45, X0/46XX (mosaic)] were observed in six patients. In a further four CEB males, one CEB female and one girl with EC, interestingly, an association with Down syndrome was found [50]. Aneuploidy of sex chromosomes in five of these cases might point to (a) gonosomal loci (locus) involved in the formation of EEC. However, the observation of different sexes in two subsequent spontaneous abortions is contradictory [54]. Structural aberrations have been identified in six EEC cases and one patient simultaneously presenting with EC and hypomelanosis of Ito. Although the exact breakpoints have not been determined in any of these cases, several translocations involving the region q32-ter on chromosome 9 were detected. Based on this observation, Thauvin-Robinet et al. [55] investigated the gene coding for steroidogenic factor 1 (*SF-1*; 9q33.3) in their patient, and the *SET *gene (suppressor of variegation, enhancer of zeste and Thrithorax; 9q34.11) has been investigated in another 33 EEC patients [56]. Although no mutation could be detected in these studies, other genes residing in this region might be involved in the etiology of the EEC. One EC patient with aminoglycoside-induced deafness, pigmentary disturbances, and spinal anomalies showed a mitochondrial 12S rRNA mutation [57].

Utsch et al. observed an *MYH9 *mutation in a patient with CEB, duplication of the vagina and congenital macrocytic thrombocytopenia [58]. Defects in *MYH9 *have been identified in macrothrombocytopenia patients [59], but there was no molecular evidence for an association with urogenital malformations. Hipp et al. compared the genetic profiles of bladder smooth muscle cells from unaffected subjects and CEB patients by microarray analysis [60]. This led to the identification of a genetic signature with 961 genes being over-expressed and 432 genes found to be under-expressed in the patients' samples. Analysis of these signatures revealed over-expression of inflammatory genes and under-expression of developmental genes, including smooth muscle genes like *GATA6 *and *connexin 43*. Whether these findings may contribute to exstrophic malformations in early embryogenesis remains to be elucidated.

### Teratogenic agents and EEC

Twin studies and epidemiological data suggest environmental factors play a role in the EEC etiology. However, the existing epidemiological studies have not identified major teratogenic factors [2,5,7]. Descriptive epidemiological and clinical data indicate male gender, race, advanced parental age [2] and increased parity, even after adjusting for age [61], as predisposing risk factors. Gambhir et al. [7] described periconceptional maternal exposure to smoking to be significantly more common in patients with EC than in a combined group of patients with E and CEB (p = 0.009). In two particular cases, each occurrence of EEC was reported after periconceptional maternal exposure to apparent infections [62,63] and to teratogens, like drugs [21,64] or alcohol [65,66]. Medications ascribed to harbor teratogenic effects have been reported rarely ["flu-shot" in one case, diphenylhydantoin, phenobarbital and mysoline in a further case [18]; diazepam [67]; misoprostol [68]; heparin [21]; valproic acid [22]. In an E associated with Al Awadi/Raas-Rothschild syndrome, exposure to X-rays was mentioned during pregnancy [69]. Several reports described the occurrence of EEC in infants resulting from *in vitro *fertilization, but it is still a matter of debate whether the incidence of EEC children conceived by *in vitro *fertilization appears to be higher than expected [70-73].

## Diagnosis

### Clinical

EEC diagnosis is usually made clinically by inspection after birth.

### Laboratory studies

EEC specific laboratory tests are not available. In EEC after birth, routine laboratory studies should include a basic metabolic panel including assessment of baseline renal function as a minimal standard before any urinary tract reconstruction. Especially in EC, an inherent short gut syndrome can result in significant electrolyte losses from the terminal ileum. Routine genetic screening of patients and parents outside of scientific studies is not yet recommended.

### Imaging studies

#### Sonography as a primary study

After birth, sonographic baseline examination of the kidneys is mandatory for all EEC patients. Later on, irrespective of the method of reconstruction, renal sonography is a perfect screening method for distinguishing any upper urinary tract changes during follow-up.

#### MRI imaging in EEC

##### a) Pelvis and pelvic floor in EEC

MRI characterized the EEC specific defect of the pelvis with an anterior pelvic outward rotation of 18°, a posterior pelvic outward rotation of 12° and a pubic rami shortening of about 30% [74,75]. In early childhood, external foot rotation of about 30° is obvious, though this rotation anomaly improves with age. A more flattened and less conical levator sling, a markedly higher coronal levator hiatus diameter and a double normal levator angle were shown after pelvic reconstruction and osteotomy in small children with EEC as compared to normal anatomy [74,75]. Additionally, in CEB the levator ani muscle is distributed more posteriorly with a shortened anterior segment in the coronal plane [74]. This specific pelvic floor defect may determine rectal prolapse, occasional anal incontinence and predispose females to uterine prolapse [26].

##### b) Genital anatomy in EEC

Pelvic MRI provides adequate information about internal genitalia before and after surgery. Therefore, MRI is mandatory before uterine prolapse repair and in complex penile redo surgery. In the literature, MRI was able to clarify the complex EEC genital anatomy in both sexes. In male EEC adults, Silver et al. documented an inherent corporal deficiency with a shorter anterior corporal body length. This deficiency contributes, in addition, to the penile chordee or bilateral penile fixation to the ascending pubic rami and to the specific short and curved penile appearance in EEC [76,77]. The development of bulbospongiosus muscle in EEC remains unclear, as only rudiments are found on the penile base. In MRI, the prostate had nearly normal volume, but even after bladder neck reconstruction did not extend circumferentially around the urethra [76]. The slightly smaller seminal vesicles had a regular shape and location [76]; fructose levels, however, were normal in over 50% of the EEC patients after functional reconstruction, indicating the fact that surgery may not severely impair seminal vesicle function [78]. Despite the complex anomaly of the penis and bladder, male EEC patients have usually normal semen production and transport from testicles to the verumontanum [1].

#### Histological findings

##### a) Of the bladder template

Histologic studies of the bladder template are not routinely performed. Although the congenital nature of the exstrophic bladder is still controversial, no fundamental histologic changes justify primary removal of the bladder template. However, investigations of the innervation and structural pattern of the exstrophic bladder showed an increased ratio of extracellular matrix to muscle content within the bladder wall, particularly a high amount of type III collagen [79-81]. As these changes seem to normalize after successful bladder closure, they might be a sign of maturation delay [79,81]. Additionally, there are urodynamic data of reconstructed exstrophic bladders showing a regular filling capacity and emptying ability [1]. In recent studies, we evaluated the distribution and density of muscarinic receptors. M3-receptor density was increased and abnormally distributed within the EEC. Density of the muscarinic receptors varied according to the number of previous operative attempts and the different subtypes of EEC [23,24]. However, although these findings may help to explain some clinical features like bladder spasm, it is not yet possible to predict the prognosis of future bladder development on the basis of pathohistological data.

##### b) Of the testis

At birth testicular histology was reported to be normal [1].

#### Follow-up studies

It is reasonable to evaluate the reconstructed bladder with a voiding cystography and urodynamic studies via a suprapubic tube. Thus, it is possible to monitor bladder storage function with sensation, detrusor activity, compliance and capacity during filling, as well as bladder emptying function with voiding and leak-point pressure and residual urine measurements. These studies provide objective evidence about outcome results after bladder neck plasty and help to avoid secondary complications as upper tract deterioration.

## Differential diagnosis

The very typical clinical picture does not implicate any further differential diagnosis.

## Antenatal diagnosis and genetic counseling

### Prenatal diagnosis

Due to high-resolution real-time ultrasound, prenatal diagnosis of EEC is usually possible between the 15^th ^and 32^nd ^week of gestation, depending on the severity of the defect and the expertise of the sonographer. The index finding is the non-visualization of a normally filled fetal bladder during repeated careful ultrasound examinations. In a retrospective review of 25 prenatal ultrasound examinations during pregnancies resulting in a newborn with CEB, a low-set umbilicus, a wide ramus pubis, diminutive genitalia and a lower abdominal mass were summarized as diagnostic key factors for EEC diagnosis, in addition to the absent bladder filling [1]. Another typical feature is a wavy cord-like segment of soft tissue protruding from the anterior abdominal wall, just below the umbilical cord insertion, strongly resembling the trunk of an elephant [1,82,83]. Gambhir et al. described that mothers of children with EC knew significantly more often prenatally that their child would have a congenital malformation than mothers of children with E or CEB did [7]. Though prenatal intervention is not necessary, early diagnosis allows optimal postnatal management. Centers that favor early closure within first hours of life, advocate scheduling of the delivery in or near a pediatric centre, familiar with optimal treatment of the severe congenital anomaly.

### Counseling of parents

After sonographic diagnosis of EEC has been established, consecutive counseling of parents must provide extensive information about the complex nature and achievable outcome of this complex anomaly. There is no doubt, that in this situation only a multidisciplinary team and members of committed self-help groups are able to support the concerned parents with their decision. Although the EEC deeply influences the psychosocial adjustment and psychosexual development of affected individuals, there is no documentation of consequences considered serious enough to recommend the termination of pregnancy after a prenatal diagnosis of CEB or E [84]. However, nowadays prenatal ultrasound diagnosis has an impact on the number of exstrophies born each year; in the series described by Cromie et al., 25% of EEC suspected pregnancies were electively terminated [83].

## Management including treatment

### Bladder exstrophy repair

Today, the functional reconstruction concept is accepted worldwide as the gold standard, though in some selected cases urinary diversion with removal of the bladder plate can be an alternative. However, both approaches have their supporters and final outcome is always a matter of appropriate patient selection, and experience of the surgeon and the whole supporting staff. Treatment strategies always need to be individualized, so that general rules can not be stated.

### Functional bladder reconstruction

The reconstructive concept is based on the fundamental agreement that the exstrophic bladder has the potential ability to gain normal bladder function as a low-pressure reservoir for urine storage, active voiding and upper urinary tract protection after functional reconstruction. Beside clinical observation, urodynamic studies reported a normal filling and emptying pattern, but impaired compliance and stability, mostly after Young-Dees-Leadbetter bladder neck reconstruction [1,24]. This bladder neck reconstruction is nowadays judged as a not nerve-sparing technique, maintaining normal detrusor function in only approximately 25% of cases [1,85]. In addition, it is an issued statement that bladder neck reconstruction has the ability, though a passive mechanism of increased subvesical resistance, to lead to complicated bladder emptying in every case [86]. Most other authors insist on the terminus continence implying a possible active, not scarred, and not obstructed emptying ability of the bladder neck region [1].

Based on retrospective studies, the primary successful operative attempt to the bladder template is claimed to be the main predictive factor for a successful outcome. Until now, quality and size of the bladder plate and its genuine influence on the outcome of a functional reconstruction is not possible to predict.

As a conclusion from previous reconstruction failures, a staged concept was derived from the studies of Jeffs and Cendron in the early 1970s. The traditional staged reconstruction popularized by Jeffs and Gearhart has been a standard approach for many years. As a modification, the so called "modern staged approach" is currently advocated by John Gearhart [1]. He made this three-stage concept - beginning with closing the bladder, the posterior urethra and the abdominal wall after pelvic ring adaptation within the first 48 hours of life - popular to many other experts worldwide [1]. The main arguments favoring early bladder closure in neonates within the first hours of life are:

1. protection of the bladder mucosa against environmental influences,

2. physiological development of bladder musculature with regular bladder cycling and

3. more virtual anatomical conditions for bladder neck and antireflux reconstruction when bladder capacity has increased.

To prevent environmental and, therefore most probably, histological metaplastic changes, protective membranes (either with topical ointment or as a plastic foil) should be applied to the bladder plate until reconstruction is scheduled. Depending on the patient age at reconstruction, the width of the symphysis diastasis and the rigidity of the pelvis during examination, osteotomy may be additionally implemented during this first procedure.

As a second step, epispadias correction follows at the age of six to nine months. In females, genital reconstruction is mostly included in the first operative procedure. As a third step, bladder neck reconstruction is only approached in those cases when bladder capacity reaches a minimum of 60 cc and, as a basic requirement, the child is ready for active participation in continence training. Due to the high incidence of bilateral reflux, antireflux plasty is always conducted simultaneously with the bladder neck reconstruction. Today, a modified Young-Dees-Leadbetter bladder neck procedure is highly recommended to protect the genuine neural supply of the bladder.

In addition to this three-stage concept, several modifications as two-stage or one-stage concepts have successfully been introduced. A modified two-stage concept is realized by Baka and colleagues, who perform a modified bladder neck reconstruction simultaneously with epispadias reconstruction at the age of four to five years after bladder closure in the newborn period [87]. The authors believe that only simultaneous reconstruction of the bladder neck and the entire urethra enables easy catheterization through a straight posterior urethra, if necessary [87]. Another two stage option is the Kelly operation, favored in Great Ormond Street and in Australia [88]. After neonatal closure, reconstruction is timed at 6 months of age, irrespective of bladder size. Detachment of the penile corpora from the inferior pubic rami and the release of the pudendal pedicle, moves the penile base freely towards the midline and therefore eliminates the effect of the pubic diastasis and brings the penis to the protruded surface. Due to inadequate urethral length, the urethra will be brought down between the corpora to a hypospadic position in most cases [88].

As a complete one-stage concept, Mitchell introduced his primary complete bladder closure with simultaneous correction of the epispadias using the penile disassembly technique [89]. Based on the hypothesis that bladder exstrophy results from anterior herniation of the bladder, the operative approach must address the bladder, bladder neck and urethra as a complete unit and move this unit permanently into the pelvis. Mitchell impressively demonstrated that penile dissection into its three components (two corpora cavernosa and the corpus spongiosum) ensures blood flow in each component and that the penis can be re-assembled in an anatomically correct configuration [89,90]. The penile disassembly technique applied simultaneously with bladder neck reconstruction however, comprises many pitfalls possibly leading to disastrous uro-genital damage when the required accuracy and expertise is not guaranteed. Since 1976, another one-stage complete reconstruction concept has successfully been realized in Germany by Schrott [91,92]. Reconstruction is timed between the eighth to tenth week of life when the baby has stabilized and all necrotic umbilical cord residues have fallen off. For the first weeks of life the bladder plate is protected with topical ointment against inflammatory and mechanical alterations. Definitive bladder size can only be estimated by sterile digital examination, detecting hidden bilateral bladder recessus during operation. Then the decision can be made whether the complete reconstruction is possible at that point. After circumcision of the bladder plate, pubovesical and pubourethral ligaments are completely divided from the ischiopubic rami down to the levator plate and caudal to the urethra ascending bilaterally from Alcock's canal to the penis or the clitoris. This complete mobilization enables anatomically correct backwards relocation of the bladder deep into the pelvis and prohibits bladder burst. An oblique incision is performed on each side up to the lateral margin between the upper and lower parts of undamaged trigone, splitting the area between the bladder, posterior urethra and the attached neurovascular bundles. The elastic trigonal muscle is tubularized for urethral prolongation and the anterior bladder wall is reinforced by a second muscular invagination. As the newborn and infant pelvis is soft enough, the symphysis is approximated in a stepwise fashion with the help of a traction bandage. Intraoperative readaptation of the symphysis pubis is secured with absorbable polydioxanone traction sutures. The advantages of every early one-stage approach are the summation of all major reconstruction steps with less scars, an unimpeded access to the bladder neck region, and a expectable rapid developing bladder capacity by rhythmic filling and passing urine against adequate resistance.

### Urinary diversion

Primary urinary diversion with removal of the bladder plate has been mainly favored by one group in Mainz in Germany [93,94]. With a normal upper urinary tract, normal serum creatinine and competent anal sphincter, a sigmoid rectum pouch is set up around the first year of life. Male genital reconstruction will either be terminated simultaneously or as a second procedure at around 1.5 years. Female genital reconstruction, as well as anterior uterus fixation, is done during the first operation [95]. The main advantages include the necessity of only a single operative procedure, the immediate creation of a low-pressure reservoir for upper urinary tract protection and the possibility to achieve usually good primary continence results.

### Male genital reconstruction

Important for reconstruction of the penis in EEC is the unique presence of two completely separated corpora cavernosa without any vascular anastomosis and the completely isolated neurovascular bundles running on the corporal outside. Regardless of the type of epispadias repair, the four following key issues must be addressed to ensure a functional and cosmetically acceptable penis:

1. correction of dorsal chordee

2. urethral reconstruction for micturition and semen transport

3. glandular reconstruction

4. penile skin closure.

To deflect the curved epispadic penis, Phillip Ransley introduced the concept of releasing dorsal chordee by incision and dorsomedial anastomosis of the corpora cavernosa above the urethra [96]. As an advancement, the Cantwell-Ransley technique completely detached the urethral plate from the corpora allowing a more effective urethral transposition below the corpora, and therefore a more effective correction of the dorsal curvature by lateral rotation of the corpora [96]. The characteristic feature of the penile disassembly by Mitchell is the complete detachment of the urethral plate from the corpora cavernosa and the glans. This technique uses the advantage of the constant blood supply of the corpora cavernosa, urethra and glans with paired dorsal arteries and neurovascular bundles to each hemiglans, the deep cavernous arteries to the corporal bodies and the spongiosus tissue to the proximal urethral plate [1,90]. After complete separation of the corporal bodies, the glans is divided, and the urethra is placed ventrally, often resulting in a hypospadic meatus requiring further repair to the penile tip [1,90]. Moreover, it has been a matter of debate whether complete penile disassembly gains as much penile length as possible complications may arise. Surgical advantages of the Cantwell-Ransley and the Mitchell techniques are the more anatomical reconstruction with only minor penile deviation and a low fistula rate due to the coverage of the neourethra through the corpora cavernosa. If the mobilization of the urethral plate from the corpora is radical enough, the corpora can most probably be joined without tension, and without corporotomy and the need for complete mobilization of the neurovascular bundles. However, scars and shortness of the neurovascular bundles may later cause severe, often uncorrectable penile deviation. As a basic requirement of all these procedures, meticulous dissection using a magnification glass is absolutely warranted to maintain blood and nerve supply and therefore avoid erectile dysfunction and corporal atrophy.

### Incontinence in isolated epispadias

Even in distal penile shaft E with only a mild genital defect, urinary incontinence occurs in up to 75% of cases. During cystoscopy, a defect of the external sphincter can be identified as a longitudinal attenuated tissue strip from the bladder neck through to the urethral sphincter. This urethral tissue must be surgically removed. The urethra must be retubularized to an adequate size and the external sphincter and the pelvic floor musculature must completely be readapted. In E with relevant urinary incontinence and a wide sphincter defect, a complete bladder neck procedure is needed; in mild defects, approximation of the pelvic floor during penile procedure might be sufficient. Very often the bladder wall is thin in E, so potential muscular support for the bladder neck is only minor and therefore operative outcome restricted. Osteotomy, however, is hardly recommended in E.

### Female genital reconstruction

Female genital anatomy in the EEC is widely judged less complex than male genital reconstruction [1,26]. However, Woodhouse developed a subtle reconstruction concept including vaginoplasty, adaptation of the clitoris, vulvoplasty, mons plasty and redistribution of hair-bearing skin [26]. Usually, in the different EEC centers, all steps are approached by the time, but the timing itself may be different. During initial reconstruction in early infancy, the roof and lateral walls of the distal urethra are excised, along with the adjacent skin and subcutaneous tissue, and tubularized over a 10 French catheter. The usually triangle-shaped area anterior to the clitoris and labia halves is excised and the defect is closed longitudinally to fuse the bifid clitoris and the labia together above the urethral meatus. Sometimes the split clitoris is usually left untouched to protect the delicate nerve supply. Skin and tissue retraction in the mons pubis area is cosmetically improved by mobilizing adjacent inguinal tissue and rotating it medially into the affected area.

Vaginoplasty is advisable in about 2/3 of the cases. To prevent repeated dilatations during childhood, episiotomy or a simple introitusplasty using a Fortunoff flap can safely be done during or just before puberty. Stein et al. perform female genital reconstruction with vaginal approach at the age of 3-4 years [95,97]. In the single-stage-Erlangen-technique adaptation of the labia and mons plasty are performed at the initial reconstruction, whereas vaginoplasty is usually done at the beginning of puberty, if necessary [92].

### Role of osteotomy

The role of osteotomy in exstrophy reconstruction has always looked towards easier symphyseal approximation, secure abdominal wall closure, placement of the entire bladder deep into the pelvis and reapproximation of the pelvic floor towards the midline. The observation that girls sometimes got dry after bladder closure and pelvic adaptation without any bladder neck surgery underlines the importance of pelvic floor restoration for continence results [1]. Interestingly, osteotomy or pelvic adaptation seems to gain importance in prevention of uterine prolapse and the overall functional outcome results [1,74,77]. Furthermore, whatever reconstructive method is used to close or adapt the pelvis in early childhood, the symphysis will always reopen over time. Unfortunately, the influence of reconstructive surgery on the pelvic soft tissue structures in adulthood is still unclear. Additionally, there is ongoing debate whether osteotomy may cause or prevent orthopedic long-term outcome problems. Indicating an osteotomy complication after, especially in adulthood, may be severe and must therefore be considered when discussing this procedure. In our experience, osteotomy is not necessary in early childhood in CEB. Nowadays, osteotomy is mandatory in EC reconstruction, due to the severe pelvic asymmetry and the large ventral defect, which should be evaluated with 3D MRI before any surgical attempt. In failed exstrophy closure, however, especially in complete bladder dehiscence or bladder prolapse, osteotomy and immobilization afterwards enables a tension-free reclosure of the anterior abdominal wall, a more effective restoration of the pelvic floor and finally improvement of continence results.

## Prognosis

### Continence results and long-term complications after functional reconstruction

Though countless publications on EEC exist, surgical outcome data have mostly been ascertained retrospectively, as single-center or single-surgeon experiences. Definitions of successful outcome, observation periods and end-points, as well as evaluation of complications and, in particular, terminology focusing on the terms "continence" or "social continence" diverge immensely. Woodhouse was the first who revealed that bladder function in EEC is not stable over time, and late failure with muscular atony may occur [98]. Nowadays, it is reasonable to expect continence rates of about 80% in childhood [1,85,86,89,96]. Within this concept, however, though most exstrophic bladders can be preserved, spontaneous voiding is not guaranteed and, especially after childhood, an increasing number of patients need bladder augmentation or self catheterization either via the urethra or via a catherizable stoma. In our first 100 one-stage functional reconstructed EEC patients, 47 underwent a primary and 53 a redo reconstruction with a mean observation period of 11.1 years [91]. Complete continence after primary reconstruction with spontaneous voiding was possible in 72.3% of the patients; whereas reliable continence dropped after redo bladder neck plasty to only 41.5% [91]. These outcome data are comparable to other high-volume EEC centers [1,87-89,91,98,99]. If primary closure fails, only 60% obtain adequate capacity for a planned bladder neck reconstruction in a staged concept. If the second closure fails, only 40% will have adequate capacity for a bladder neck reconstruction and only 20% will become dry [100]. Numerous possible complications (such as recurrent urinary tract infections, recurrent epididymitis, residual urine and therefore urinary calculi formation, *etc*.) may complicate the course of the disease and require meticulous long-term care.

### Reconstruction failure after functional reconstruction

Reconstruction failure is usually assessed clinically, by endoscopy and with urodynamics. Identifying the medical problem, with simultaneous consideration of the individual and family history, should lead to further therapeutic recommendations. If bladder storage is impaired, the bladder can be augmented with bowel, preferentially with ileum or sigma. After augmentation, sufficient bladder emptying must be provided either through catheterization per urethram or through a catheterizable channel according to the Mitrofanoff principle. If the bladder neck resistance is low, injectable materials like dextranomer/hyaluronic acid can enforce urethral resistance [101]. This minimally invasive approach allows quite reasonable success in order to improve continence, but success will be only durable after at least 3 injections [101]. A definitive solution is bladder neck closure with creation of a catheterizable channel, but reliable compliance of patients and parents are of fundamental importance for success. In cases with bad bladder development, upper tract deterioration and continence is not achievable over a reasonable period and a well-balanced benefit-effort-analysis urinary diversion should be performed. Patient age, social background and life style should be taken into consideration to decide whether a catheterizable pouch or a sigma-rectum-pouch is chosen for urinary diversion.

### Continence results and long-term complications after urinary diversion

In the literature, urinary diversion provides very high primary continence rates. Thirty eight children with a mean age of 5 years were reported to be completely continent during the day and only 8.6% used pads during the night [93,94]. However, gaining anal continence in childhood after urinary diversion is an individual process until the child is about 5-7 years old. Another advantage of this method is the fact that the upper urinary tract is protected due to the modified low-pressure reservoirs. Using the new antirefluxive ureteral implantation techniques: 15.8% had episodes of pyelonephritis, and 14.5% needed ureteral reimplantation (due to stenosis in 10.1% and reflux in 4.4%) [94]. Sixty nine percent of patients need alkalizing drugs to prevent hyperchloremic acidosis, and therefore potentially impaired bone mineralization and growth deficiency. On the other hand, severe long-term complications must be considered like the development of adenocarcinoma at the ureterointestinal anastomosis after 15-25 years. The incidence of these mostly adenocarcinomas has been estimated to be 3.5 up to 19% [102], and is 8-550 times more frequent in patients with ureterosigmoidostomy compared with the incidence of colorectal cancer in age-matched controls. Recent data showed that colonic adenomas can be securely managed with local excision and during the observation period no recurrence occurred as yet. Therefore, annual rectoscopy is highly recommended after the 10th postoperative year [102].

### EEC long-term outcome issues: fertility, psychosocial and psychosexual outcome

#### Male EEC patients: fertility and genital outcome

Nowadays, modern reconstruction techniques enable acceptable functionality and cosmetics in the EEC. Current and future efforts reflect that congenital genitourinary anomalies have tremendous impact on adult life [84,97-99,103-106]. A fulfilled sexual life, being married and having offspring represent main indicators for a successful genital rehabilitation [78,99,103-106]. Naturally, interest in sexual activity is normal. Most striking for the male EEC patients are penile size and deviation, as well as anxiety about and avoidance of sexual interaction. Despite these severe restrictions, about 50% of male EEC patients practice sexual intercourse. A positive attitude towards micropenis and the male gender role can be achieved by patients and parents, but mental success mainly depends on parental enthusiasm, openness, and sufficient knowledge about the anomaly. Due to these restrictions, close and long lasting relationships were the consequence [78,84,103-106]. In his literature review, Woodhouse found at least some kind of ejaculation in 75% of the EEC patients, regardless of the reconstruction method, and concluded that about 50% of the male EEC patients were able to father children [103]. Recent long-term results regarding fertility in the EEC do sparsely exist [46]. There is no consent as to whether primary diversion or functional reconstruction will allow better semen transport or fertility [1,78,193,104]. Complications of reconstructive surgery and postinfectious effects, however, seem to be disastrous to fertility in male EEC patients. Recently, incidence of primary spermatogenesis failure, especially in the azoospermia group, was reported to be about 20% [78]. Therefore, pathogenesis of the impaired fertility in EEC is probably multifactorial [78]. Our long-term data suggest that functional bladder neck reconstruction with a consequent anatomical placement of the colliculus seminalis in the posterior urethra, however, allows antegrade ejaculations in 94.1% of the patients [78]. So not only for continence, but also for ejaculation and fertility, the primary successful and anatomically correct approach to the bladder neck seems to be the key point.

#### Female EEC patients: fertility and genital outcome

In general, female EEC patients require comparatively little surgery with mostly acceptable cosmetic outcome. Due to normal internal genitalia, mainly not affected by the reconstructive bladder surgery, fertility should usually be normal. Furthermore, as a consequence of a low cervical insertion an even a higher chance for pregnancy is assumed. Additionally, Woodhouse stated that, compared to males, female exstrophy patients have fewer problems with sexuality and sexual intercourse [26]. Thirty four of his 42 female patients were able to participate in sexual intercourse; 12 of them did not even require vaginoplasty; and 32 were married or maintained a steady partnership. In this group, 22 pregnancies resulted in 19 healthy babies; only three pregnancies were terminated for therapeutic reasons not related to EEC [26]. Stein reported 14 adult female patients older than 18 years after urinary diversion: 93% were married; only 3 reported unpleasant sexual activity [97]. However, Matthews reported a series of 83 female EEC patients, who had a late onset of sexual activity with a mean age of 20.2 years despite appropriate sexual desire [106]. Six patients complained about dyspareunia; five refused sexual intercourse because of unsatisfying cosmetics; and only 12 experienced orgasms [106]. Due to the less complex female reconstruction, comparatively little attention is drawn to the outcome and so, unsatisfactory reconstructed genitalia often impair female self-esteem [105]. Thus, gender-related outcome seems to be of fundamental impact and warrants physicians' empathy and commitment [107]. However, in adulthood, vaginal or uterine prolapse is the most striking problem. Still, there is a paucity of knowledge about pelvic floor anatomy after reconstruction and sparse reports have failed to determine risk factors for this major complication. Inadequate pelvic ring adaptation and therefore pelvic floor adaptation in combination with removal of the bladder template may be risk factors for uterine prolapse [1,108]. More recently, there is some evidence that restoration of the pelvic floor and therefore pelvic adaptation or osteotomy might prohibit uterine prolapse [108]. Established treatment strategies of uterine prolapse include sacrofixation, uteri- or hysterectomy. Only sparse long-term data exist, but benefit in our experience is only for short periods. Complications like vault prolapse occur, and this might be a result of the finally unclear pathophysiology.

### Psychosocial and psychosexual outcome in both sexes

Most available data about psychosocial and psychosexual development in EEC refer to well-adjusted adults who have already passed through puberty and adolescence. Standard questionnaires provide evidence for a normal quality of life, a usually high social adaptation level with good school performance and education standards. In adulthood, many EEC patients have a so-called ordinary life including marriage, sexual relationships, family relations, children of their own, and professional success. Some of our own adult patients, however, express their wish to erase the memory of those challenging times and complain about loneliness in certain periods of life (*e.g*., puberty). Health status was usually derived from continence status. Impairment of daily life and self-esteem is common in 25% of cases, contacts with peers were present, but a lot effort was put into hiding the anomaly in daily life [105,107]. EEC patients themselves stated that openness about the EEC, regular upbringing, sufficient information, and a supportive parental attitude regarding self-esteem and autonomy as the best strategies for successful coping. Predictive factors for mental health were parental warmth, urinary continence and genital appearance [1]. Hence, some reports state a certain prevalence of psychiatric diagnoses consisting mainly of internalized conflicts and emotional problems such as marked anxiousness, sadness, depression, low self-esteem, poor body concept, isolation and withdrawal, others deny the evidence of psychopathology in relation to EEC [1]. Attainment of continence at a later age consequently leads to more externalized struggles with low adaptive behavior scores. Due to their specific developmental implication, genitourinary malformations may create vulnerabilities to psychosexual dysfunction due to prolonged incontinence, residual genital defects and postsurgical genital appearance [1]. Continence is often achieved by several operations, and is not a result of learning and developing processes [1]. Parental overprotection and physical handicaps like incontinence - sometimes present until late school age - may hold back children at school or during social activities with peers.

For the parents, the first year of life of the EEC child is a major challenge, sometimes with definite impairment of the child-parent relationship and severe problems with parental coping strategies. Thus, parents should be offered psychological support as soon as possible. As a consequence, support from a multidisciplinary team, helping these affected individuals and parents through the whole of childhood and adolescence, is mandatory. A prospective analysis of clinical predictive factors in gender-related long-term outcome is needed to provide an individualized flexible treatment strategy with predictable success and quality of life. Besides the pediatric urologist, this must also comprise the pediatric orthopedic surgeon, the pediatrician, the pediatric psychologist experienced in urology, experienced pediatric nurses and urotherapists.

### Risk of malignancy in the exstrophic bladder

At birth, hamartomatous polyps are visible on the exstrophic bladder surface in about 50% of the cases [109]. These polyps have been interpreted as reactive, potential pre-malignant environmental changes. Therefore, closure of the bladder template within the first few hours of life is widely recommended. However, no direct proof was made that bladder cancer is definitely developing from a polyp or a coexistent glandular metaplasia [109]. After several operative attempts to the bladder, epithelial damage in terms of glandular cystitis or intestinal metaplasia was more commonly found within the EEC [109]. Until now, natural history of this intestinal metaplasia is still unclear and cannot be ruled out as a strong risk factor for adenocarcinoma or other urothelial malignancy in long-term follow-up [109]. There are some reports about adenocarcinomas and squamous cell carcinomas occurring in unreconstructed, environment-exposed exstrophic bladders [110,111]. Astonishingly, neoplasia was found in the exstrophic bladder remnant, even when early cystectomy had been performed [110]. So, the estimated risk for bladder carcinoma in the EEC population was 700 times higher than the age-matched general population [110].

### Unresolved questions

Taking all treatment perspectives together, the most serious problem is the lack of any histological or clinical data allowing a reliable prognosis of future bladder growth and long-term storage and voiding function after birth. Therefore, the outcome and outcome-related prognostic factors are still unclear. Prospective outcome analysis is mandatory to further improve treatment strategies. In addition, current long-term outcome analysis now allows judgments to be made about treatment strategies implemented 20-30 years ago. A standardized follow-up program as a result of long-term outcome studies will definitely help to improve the final results and therefore lifelong outcome success.

## Abbreviations

EEC: Exstrophy-epispadias complex; E: Epispadias; CEB: Classical bladder exstrophy; EC: Exstrophy of the cloaca; OEIS complex: omphalocele, exstrophy, imperforate anus and spinal defects; MRI: Magnetic resonance imaging.

## Consent

Written informed consent was obtained from the parents for publication of accompanying images of BEEC patients. A copy of the written consent can be made available for review by the Editor-in-Chief of this journal.

## Competing interests

The authors declare that they have no competing interests.

## Authors' contributions

All authors contributed to this review article according their scientific interests and activity. AKE and WHR: clinical parts like definition, clinical description, diagnosis, management including treatment, prognosis. HR and ML: epidemiology, etiopathogenesis, antenatal diagnosis and genetic counseling
